# The Composition and Phosphorus Cycling Potential of Bacterial Communities Associated With Hyphae of *Penicillium* in Soil Are Strongly Affected by Soil Origin

**DOI:** 10.3389/fmicb.2019.02951

**Published:** 2020-01-08

**Authors:** Xiuli Hao, Yong-Guan Zhu, Ole Nybroe, Mette H. Nicolaisen

**Affiliations:** ^1^Section for Microbial Ecology and Biotechnology, Department of Plant and Environmental Sciences, University of Copenhagen, Copenhagen, Denmark; ^2^Key Lab of Urban Environment and Health, Institute of Urban Environment, Chinese Academy of Sciences, Xiamen, China; ^3^State Key Laboratory of Agricultural Microbiology, Huazhong Agricultural University, Wuhan, China; ^4^Key Laboratory of Arable Land Conservation (Middle and Lower Reaches of Yangtze River), Ministry of Agriculture, College of Resources and Environment, Huazhong Agricultural University, Wuhan, China; ^5^State Key Lab of Urban and Regional Ecology, Research Center for Eco-Environmental Sciences, Chinese Academy of Sciences, Beijing, China

**Keywords:** *Penicillium*, soil microcosm, hyphae-associated bacterial community, soil origin, phosphorus cycling gene

## Abstract

Intimate fungal-bacterial interactions are widespread in nature. However the main drivers for the selection of hyphae-associated bacterial communities and their functional traits in soil systems remain elusive. In the present study, baiting microcosms were used to recover hyphae-associated bacteria from two *Penicillium* species with different phosphorus-solubilizing capacities in five types of soils. Based on amplicon sequencing of 16S rRNA genes, the composition of bacterial communities associated with *Penicillium* hyphae differed significantly from the soil communities, showing a lower diversity and less variation in taxonomic structure. Furthermore, soil origin had a significant effect on hyphae-associated community composition, whereas the two fungal species used in this study had no significant overall impact on bacterial community structure, despite their different capacities to solubilize phosphorus. However, discriminative taxa and specific OTUs were enriched in hyphae-associated communities of individual *Penicillium* species indicating that each hyphosphere represented a unique niche for bacterial colonization. Additionally, an increased potential of phosphorus cycling was found in hyphae-associated communities, especially for the gene *phnK* involved in phosphonate degradation. Altogether, it was established that the two *Penicillium* hyphae represent unique niches in which microbiome assemblage and phosphorus cycling potential are mainly driven by soil origin, with less impact made by fungal identity with a divergent capacity to utilize phosphorus.

## Introduction

In the dynamic soil environment, highly diverse bacterial and fungal communities co-exist (Nazir et al., 2013). Although there is competition between fungi and bacteria for soil resources, the presence of fungal hyphae provides colonization sites for bacteria, and creates new microhabitats with abundant and available carbon substrates (de Boer et al., 2005; Halsey et al., 2016). Approaches to identify bacteria enriched by the presence of fungal hyphae include studies of soil affected by hyphae compared to hyphae-free soils (Nazir et al., 2010, 2013; Nuccio et al., 2013; Wang et al., 2016), as well as baiting approaches for the identification of bacteria colonizing fungal hyphae in laboratory (Scheublin et al., 2010) or close-to-natural soil conditions (Ghodsalavi et al., 2017). According to these studies, environmental differentiation between the hyphosphere and the bulk soil leads to different compositions of hyphae-associated and bulk soil bacterial communities, with genera from β-Proteobacteria, γ-Proteobacteria, and Firmicutes commonly enriched in the hyphosphere or soil influenced by fungal hyphae.

Despite increasing knowledge of the composition of hyphae-associated microbiomes, the factors affecting assembly of these hyphae-associated bacterial communities remain elusive. Ballhausen et al. (2015) found differences between bacteria adhering to hyphae of *Trichoderma*, *Mucor*, and *Rhizoctonia* exposed to bacteria extracted from rhizosphere soil, while weaker effects of the identity of four arbuscular mycorrhizal fungi (AMF) on hyphae-associated bacterial communities have been observed using a related approach (Scheublin et al., 2010). In more realistic soil systems, the identity of AMF and ectomycorrhizal fungi has been shown to influence the composition of bacterial communities associated with hyphal bundles or ectomycorrhizal roots (Boersma et al., 2009; Marupakula et al., 2016). Abiotic factors of the soil environment could also be important for shaping fungal-associated bacterial communities (Filonow and Arora, 1987; Rousk et al., 2009), as shown for ectomycorrhizal roots where soil pH, soil carbon (C), nitrogen (N) and phosphorus (P) content as well as C:N ratios have a strong effect on bacterial communities (Simon et al., 2016; Marupakula et al., 2017).

Fungal hyphae are not only the perfect sites for bacterial colonization and dispersal, but also serve as hotspots for fungal-bacterial interactions and nutrient cycling (Furuno et al., 2012). In the hyphosphere, carbon flow from fungal hyphae to hyphae-associated bacteria could occur through the consumption of hyphal exudates, grazing on living hyphae or degradation of senescent hyphae (Drigo et al., 2010; Jansa et al., 2013). From the perspective of mutualistic interactions, bacteria attracted by hyphae could supply fungi with nutrients such as N and P. Previous studies have shown that fungi select bacterial communities with a higher efficacy for utilizing fungal-derived carbon compounds and also with a greater potential for mineral weathering (Uroz et al., 2007, 2011). Another type of evidence of hyphae serving as hotspots is the frequent appearance of N-fixing bacteria or P-solubilizing bacteria (PSB) enhancing the capacities of nutrient cycling in the hyphosphere (Zhang et al., 2014; Zagryadskaya, 2017). In relation to P turnover, Zhang et al. (2014) demonstrated that the high C:P ratio in the hyphosphere may stimulate the recruitment of bacteria with an enhanced ability to mobilize soil (in)organic P, while other studies suggest competition for P between mycorrhizal fungi and PSB in the hyphosphere soil (Brooks et al., 2011; Wang et al., 2016).

*Penicillium* fungi are ubiquitous in soil (Egbuta et al., 2016) and are considered a key group in P cycling (Harvey et al., 2009; Richardson et al., 2011). In part this is due to their ability to solubilize inorganic P through the release of organic anions (Richardson et al., 2011), although with considerable differences between species (Wakelin et al., 2004). Elite strains have been developed into commercial biofertilizer inoculants for the improvement of plant P nutrition (Richardson et al., 2011). However, for *Penicillium*, the effects of soil conditions and fungal identity on bacterial community composition and their potential role in P cycling are largely unknown.

The objectives of the current study were therefore to investigate the impacts of soil origin on (1) the community structure and (2) the P-cycling potential of hyphae-associated bacteria, assembled on two *Penicillium* isolates with different P-cycling potential. Baiting microcosms with P-solubilizing *P. canescens* or non-solubilizing *P. janthinellum* were established to recover hyphae-associated bacteria under close-to-natural conditions. Soils differing in physicochemical properties, including P availability, were used to constitute a diverse base for hyphal selection of bacterial communities.

## Materials and Methods

### Soil Microcosm Setup and Sample Collection

*Penicillium canescens* ATCC 10419 (P_c_) and *Penicillium janthinellum* ATCC 10455 (P_j_) were originally isolated from soil in the United Kingdom and Nicaragua respectively. The strains differ in their P-solubilizing capacities. P_c_ produced 1,343 ± 40 mg/L PO${}_{4}^{3-}$ in liquid culture with 5.4 g/L hydroxyapatite, while almost no solubilized phosphorus was determined for P_j_ (0 ± 21 mg/L ${\text{PO}}_{4}^{3-}$). Strains were grown for 14 days at room temperature with shaking at 300 rpm, in a medium containing: Sucrose 10.0 g/L, NH_4_Cl 0.4 g/L, KNO_3_ 0.78 g/L, NaCl 0.1 g/L, MgSO_4_⋅7H_2_O 1.0 g/L, CaCl_2_⋅2H_2_O 0.1 g/L, Hydroxyapatite 5.41 g/L. At this time, soluble ${\text{PO}}_{4}^{3-}$ was measured by the BioVision Phosphate Colorimetric Assay (BioVision, Mountain View, CA, United States) (data kindly provided by Drs. Dave Greenshields and Michael Furlan, United States).

To ensure distinct bacterial communities for hyphal recruitment, five soil samples from different geographical locations, which had received either inorganic fertilizer or organic manure amendments, and differing in P availability, pH and texture, were used in this study. S_1_ was sampled from the LTNDT (Long-Term Nutrient Depletion Trial) N_1_P_1_K_1_ field at University of Copenhagen, Denmark (Van der Bom et al., 2017). S_2_ and S_3_ were sampled from the topsoil (5–15 cm) and subsoil (30–45 cm), respectively, of fields amended with animal manure at Sjaellands Odde in northern Zealand, Denmark (Paulin et al., 2013). S_4_ was obtained as S_3_ soil with the application of 68 mg/kg P in the form of NaH_2_PO_4_⋅H_2_O just before incubation of fungal hyphae. S_5_ was sampled from topsoil at the Jyndevad agricultural research station in southern Jutland, Denmark (Paulin et al., 2013). Soil characteristics are listed in Supplementary Table S1. Before setting up the soil microcosms, soil water content was adjusted to 14.1% and soils were pre-incubated at 26°C for 8 days to activate soil microorganisms.

Soil microcosms were set up as described previously (Ghodsalavi et al., 2017). Briefly, 2 × 10^5^ spores of *P. canescens* (P_c_) or *P. janthinellum* (P_j_) were plated on 1/5 strength Potato Dextrose Agar with 2% agar (PDA, Difco Laboratories, Detroit, MI, United States), and incubated at 26°C overnight. Fungal plugs were placed on sterilized glass slips (25 mm, VWR International, Bridgeport, NJ, United States) with the fungal hyphae facing down. The slips were placed on water agar plates. After 3 days’ incubation at 26°C, the fungal plugs were removed and the glass slips covered with fungal hyphae were transferred to sterile polyamide mesh bags (50 μm, Sintab Produkt AB, Sweden). Mesh bags with hyphae-covered glass slips were buried in the middle of a Petri dish filled with 90–100 g of soil, and incubated at 26°C. Glass slips were recovered from soil microcosms and washed twice gently using 500 μl MilliQ water to remove the free-living bacteria. For DNA extraction, *Penicillium* hyphae and associated bacteria on glass slips were subsequently harvested by scraping off hyphae with a scalpel while washing with sterilized MilliQ water. Biological triplicates were collected for DNA extraction, where the suspensions from 10 glass slips were pooled as one replicate. Soil microcosms without glass slips were processed as described above and used for soil DNA extraction. Sterilized glass slips without fungal inoculum were used as negative controls.

### Hyphal Viability and Bacterial Colonization

Glass slips covered with growing hyphae were collected from soil microcosms after 0, 4 and 8 days’ incubation, and used to check hyphal structure and viability. Calcofluor^®^ White M2R (CFW) and the FUN^®^ 1 from LIVE/DEAD^®^ Yeast Viability Kit (Molecular Probes, Eugene, OR, United States) were used separately since the interference of CFW and FUN 1 staining was observed for *Penicillium* hyphae. To stain fungal cell wall chitin, 30 μl of 25 μM CFW was added on top of the slips to flood fungal hyphae, and incubated in the dark at room temperature for 15 min. Hyphae flooded with 25 μM FUN 1 were incubated at 30°C in the dark for 1 h before checking the metabolic state of the hyphae. To detect bacterial attachment at day 8, 20 μl of SYBR Green solution (100 times dilution of the stock, Invitrogen Life Technologies, Carlsbad, CA, United States) was added and incubated at room temperature for 5 min. Each staining process was carried out independently, and at least two glass slips were processed for each fungal species. Stained hyphae, spores and bacteria were observed using an epifluorescence microscope (Axioskope 2, Zeiss, Oberkochen, Germany) equipped with a FITC filter (Exciter filter BP 450–490 nm, emission long pass LP 520 nm) and a DAPI filter (Exciter filter G 365 nm, emission long pass LP 420 nm) for FUN 1, SYBR Green, and CFW staining respectively. All images were observed under a 63 × oil objective lens (Carl Zeiss), captured with a Zeiss AxioCam digital camera, and processed with AxioVision Software and the ImageJ program.

### DNA Extraction, Sequencing, and Data Analysis

Soil DNA was extracted from 0.25 g microcosm bulk soil using the PowerSoil^®^ DNA Isolation Kit (MoBio Laboratories, Inc., Carlsbad, CA, United States). DNA from the suspensions of *Penicillium* hyphae and associated bacteria were extracted using the DNeasy Blood & Tissue kit (Qiagen, Santa Clarita, CA, United States) following the protocol for Gram-positive bacteria. The 16S rRNA gene V_3_–V_4_ region was amplified using primers Bakt_341F (CCTACGGGNGGCWGCAG) and Bakt_805R (GACTACHVGGGTATCTAATCC), purified and sequenced by the Illumina MiSeq platform as described previously (Ghodsalavi et al., 2017). Raw sequence data were submitted to the GenBank Sequence Read Archive with SRA accession: SRP132339.

The paired-end reads (>200 bp) were merged and quality screened using PANDAseq with an assembly quality score of 0.9 (Masella et al., 2012), generating 1,904,155 merged sequences with 462 ± 6 bp. Downstream sequence analysis was processed using the QIIME pipeline, including *de novo* OTU (Operational Taxonomic Unit) clustering by UCLUST (97% similarity threshold), representative sequence picking, and taxonomic assignment by UCLUST against Greengenes 13_8 (Caporaso et al., 2010). The OTU table was filtered to exclude singletons and taxa identified as Chloroplasts and Mitochondria. Phyloseq files were generated using R version 3.3.2 with the phyloseq package (McMurdie and Holmes, 2013), and further used to calculate alpha diversity indexes after rarefaction to an even depth of 8,903 reads. NMDS (non-metric multidimensional scaling ordination) and PERMANOVA (permutational multivariate analysis of variance, 10,000 permutations) were performed based on a weighted UniFrac distance of OTU matrix using vegan package in R. Hierarchical cluster analysis (Ward’s method) was performed based on the Bray–Curtis dissimilarity using the mean relative abundances of bacterial OTUs at phylum and genus level. To identify taxonomic biomarkers with significant differential abundance among interested samples, LEfSe (linear discriminant analysis effect size) was performed using Kruskal-Wallis and pairwise Wilcoxon tests at a significance level of *P* < 0.05 (Segata et al., 2011). The effect size of differentially abundant taxa was estimated by LDA (linear discriminant analysis), with the logarithmic LDA score above 2 as the threshold for discriminative features. All LEfSe analysis was performed using the Galaxy framework^1^. Identification and visualization of differentially abundant OTUs enriched in the soil, P_c_ and P_j_ hyphae were performed in R using the code published by Zgadzaj et al. (2016), where a negative binomial GLM (generalized linear model) was employed with a false discovery rate (FDR) < 0.05, and on the OTUs with relative abundance above 0.5 ‰. Variance partitioning and partial redundancy analysis (pRDA) were carried out using vegan package in R to explain the contribution of soil conditions and fungal identities to the variation in hyphae-associated bacterial communities. Mantel test was used to explore the correlation between the Euclidean distance of edaphic factors and Bray–Curtis dissimilarity of OTU matrix from hyphae-associated bacterial community in R (vegan package). To determine the relationships between relative abundance of bacteria phyla and edaphic factors, redundancy analysis (RDA) was applied using Canoco version 4.5. Student’s *t*-test was used to determine the significant difference in relative abundance of major phyla/genera with relative abundance above 2%, between soil and hyphae-associated samples. P cut-off values were adjusted to account for multiple testing. One-way ANOVA followed by the Tukey’s HSD test and Pearson correlation analysis was performed in SPSS Statistics 22 (IBM). Networks of significant positive correlations (*P* < 0.05) were visualized using Cytoscape 3.5.1 (Shannon et al., 2003). All graphs were annotated using Adobe Illustrator (Adobe Systems, United States).

### Quantification of Phosphorus Cycling Genes

16S rRNA genes and genes involved in phosphorus cycling (*bpp*, *phoX*, *phoD*, *phnK*, *ppX*, and *pqqC*) were quantified by qPCR using the Mx3000P qPCR system (Agilent Technologies, Santa Clara, CA, United States). Twenty-μl reaction mixtures were prepared with 10 μl Brilliant III Ultra-Fast SYBR^®^ Green Low ROX qPCR Master Mix (Agilent Technologies, Santa Clara, CA, United States), 1 mg/ml BSA (bovine serum albumin, Thermo Fisher Scientific, Waltham, MA, United States), 0.4 μM of each primer, and 2 μl of template DNA (1 to 10 ng/μl). Thermal cycling was as follows: an initial cycle of 95°C for 3 min, followed by 40 cycles each of 95°C for 20 s and 58°C for 30 s. A dissociation curve was generated at the end of the qPCR program to verify a specific melting temperature of the amplified product, by including a cycle of 95°C for 1 min, 55°C for 30 s, and finally reaching 95°C by increments of 0.5°C s^–1^, each followed by a fluorescence acquisition step. Primers used for PCR reactions are listed in Supplementary Table S2. Absolute abundance was calculated based on a standard curve for each target gene. Standard plasmids with the insertion of target genes were constructed using the TOPO^®^ TA Cloning^®^ Kit (Invitrogen, United States). Ten-fold serial dilutions of standard plasmid were included in each qPCR run for each target gene. Detailed descriptions on plasmid construction and calculations can be found in Supplementary Material. The abundance of functional genes was finally normalized to the 16S rRNA gene to calculate the normalized relative abundance (copy numbers per 16S rRNA gene). Student’s *t*-test was used to analyze significant differences in relative abundance of P-cycling genes between soil and hyphae-associated samples (*P* < 0.05).

## Results

### Hyphal Growth, Viability, and Colonization by Bacteria in Soil

**FIGURE 1:**
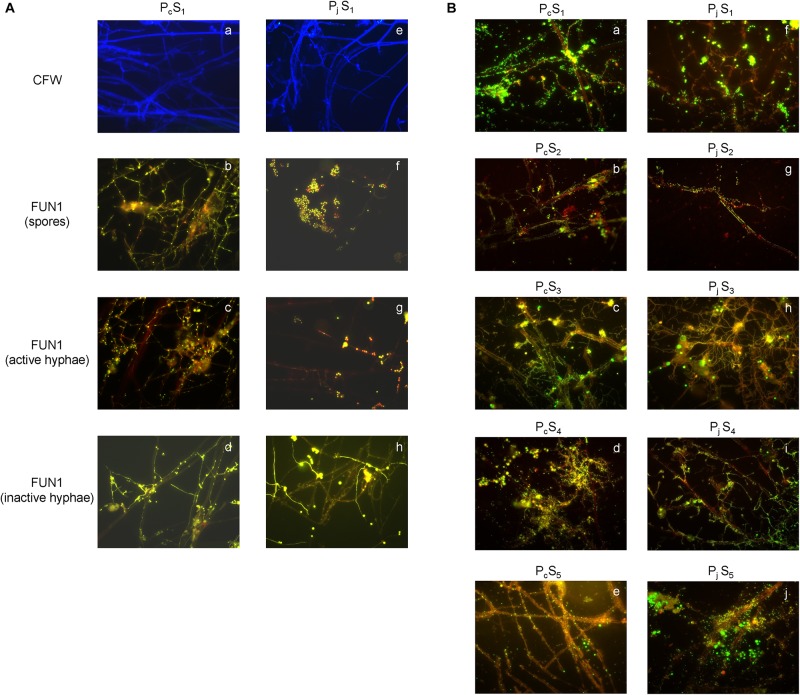
Microscopic validation for hyphal viability and bacterial colonization. **(A)** The structure and viability of hyphae from *P. canescens* (P_c_) and *P. janthinellum* (P_j_) located on the surfaces of glass cover slips after 8 days’ incubation in S_1_ microcosms. **(a,e)** Calcofluor White (CFW)-stained hyphae for P_c_ and P_j_; **(b,f)** mixture of both metabolically active and inactive spores for P_c_ and P_j_; **(c,g)** FUN 1-stained metabolically active hyphae with red-orange intravacuolar structures for P_c_ and P_j_; **(d,h)** FUN 1-stained metabolically inactive/dead hyphae with diffuse green staining for P_c_ and P_j_. **(B)** SYBR Green stained bacterial colonization on *P. canescens* **(a–e)** and *P. janthinellum* hyphae **(f–j)** in five different soil microcosms (S_1_–S_5_). Both P_c_ and P_j_ hyphae were densely colonized by bacteria after 8 days’ incubation. All images were observed using a 63 × oil objective lens (Carl Zeiss, total magnification was 630x).

The presence of *P. canescens* (P_c_) and *P. janthinellum* (P_j_) hyphae on the surface of cover slips was observed during incubation lasting 8 days in S_1_ microcosms (Figure 1A and Supplementary Figure S1). For P_c_, germination of spores led to a large amount of thin, metabolically active hyphae forming a densely cross-linked network with old hyphae at day 8 (Figures 1A–b,c). For P_j_, a mixture of metabolically active and inactive spores was observed, most of which did not germinate even after 8 days (Figures 1A–f). Most old hyphae were found to be inactive/dead (Figures 1A–h), however active hyphae were observed in P_j_ samples by day 8 (Figures 1A–g). Taken together, a mixture of metabolically active and inactive hyphae and spores was observed for both *Penicillium* species, with hyphae and spores of P_c_ appearing more active than those from P_j_. Despite differences in fungal activity, both P_c_ and P_j_ hyphae were densely colonized by bacteria with various morphologies and growth forms (aggregates, chains, biofilms) after 8 days of incubation in all five soils employed in the study (Figure 1B). Control glass slips without fungal hyphae showed only minor colonization by soil bacteria (data not shown), as also previously observed (Ghodsalavi et al., 2017).

### Diversity of Soil and Hyphae-Associated Bacterial Communities

Hyphae-associated bacterial communities were compared to soil communities for five different soils (Table 1). Soils included three top soils (S_1_, S_2_, and S_5_) with different texture, pH and soil nutrient contents; further two sub-soils related to the S_2_ top soil were included, one of which (S_4_) had received an amendment with inorganic P in the laboratory prior to establishment of soil microcosm (Supplementary Table S1). Bacterial communities associated with hyphae had significantly lower OTU numbers (decreased by 69 ± 19%) and less diversity than soil communities according to both Chao 1 richness and Shannon diversity indices (Table 1). However, neither index varied significantly between P_c_ and P_j_ hyphae-associated samples in any soil. Additionally, P addition to the S_3_ subsoil did not significantly affect the richness and diversity of the soil (S_3_ vs. S_4_, *P* > 0.05) or hyphae-associated bacterial communities (P_c/j_S_3_ vs. P_c/j_S_4_, *P* > 0.05).

**TABLE 1:** Origin, sequencing information, and alpha diversity of samples used in this study.

**Sample**	**Origin**	**Soil microcosms**	**No. of sequences**	**No. of all OTUs**	**Decreased OTUs (%)^a^**	**No. of shared OTUs with soil^a^**	**Percentage of shared OTUs with soil (%)^a^**	**Shannon**	**Chao1**
S_1_	Soil 1	LTNDT N_1_P_1_K_1_	34,684 ± 566	5,873 ± 88^D^	−	−	−	6 ± 0^B^	4,844 ± 57^C^
S_2_	Soil 2	Sjaelland Odde Org.A	48,622 ± 1,794	10,035 ± 186^A^	−	−	−	7 ± 0^A^	7,349 ± 106^A^
S_3_	Soil 3	Sjaelland Odde Org.B	42,113 ± 1,092	8,269 ± 193^B^	−	−	−	7 ± 0^A^	6,048 ± 220^B^
S_4_	Soil 4	Sjaelland Odde Org.B + 60 mg/kg P (NaH_2_PO_4_⋅H_2_O)	40,130 ± 1,086	8,145± 149^rmB^	−	−	−	7 ± 0^A^	6,262 ± 172^B^
S_5_	Soil 5	Jyndevad A	38,111 ± 3,890	7,052 ± 701^C^	−	−	−	7 ± 0^AB^	5,577 ± 551^BC^
P_c_S_1_	P_c_ Hyphae	P_c_ + LTNDT N_1_P_1_K_1_	61,095 ± 3,704	3,003 ± 294^EF^	49	411	7	4 ± 0^E^	2,076 ± 103^DE^
P_j_S_1_	P_j_ Hyphae	P_j_ + LTNDT N_1_P_1_K_1_	51,560 ± 2,302	2,858 ± 244^FG^	51	468	8	4 ± 0^DE^	2,126 ± 223^DE^
P_c_S_2_	P_c_ Hyphae	P_c_ + Sjaelland Odde Org.A	22,151 ± 3,202	1,455 ± 304^H^	86	425	5	5 ± 0^CD^	1,247 ± 202^EF^
P_j_S_2_	P_j_ Hyphae	P_j_ + Sjaelland Odde Org.A	24,937 ± 4,074	1,842 ± 264^GH^	82	477	5	5 ± 0^CD^	1,581 ± 205^EF^
P_c_S_3_	P_c_ Hyphae	P_c_ + Sjaelland Odde Org.B	19,320 ± 4,167	1,255 ± 260^H^	85	354	4	4 ± 0^E^	1,110 ± 216^F^
P_j_S_3_	P_j_ Hyphae	P_j_ + Sjaelland Odde Org.B	20,669 ± 6,349	1,295 ± 310^H^	84	338	4	4 ± 0^E^	1,169 ± 249^F^
P_c_S_4_	P_c_ Hyphae	P_c_ + Sjaelland Odde Org.B + 68 mg/kg P (NaH_2_PO_4_⋅H_2_O)	21,828 ± 4,759	1,106 ± 111^H^	45	1530	22	4 ± 0^E^	938 ± 41^F^
P_j_S_4_	P_j_ Hyphae	P_j_ + Sjaelland Odde Org.B + 68 mg/kg P (NaH_2_PO_4_⋅H_2_O)	25,215 ± 1,081	1,537 ± 42^H^	43	1863	26	4 ± 0^E^	1,294 ± 91^EF^
P_c_S_5_	P_c_ Hyphae	P_c_ + Jyndevad A	49,682 ± 2,796	3,851 ± 118^EF^	86	251	3	5 ± 0^C^	2,709 ± 103^D^
P_j_S_5_	P_j_ Hyphae	P_j_ + Jyndevad A	48,555 ± 2,323	4,008 ± 117^E^	81	346	4	5 ± 0^C^	2,885 ± 43^D^

*(1) P_c_ and P_j_ are Penicillium canescens and Penicillium janthinellum respectively. All data are presented as the mean ± standard deviation of three replicates. Different superscript capital letters represent significant difference among samples (one-way ANOVA, P < 0.01). (2) ^a^Compared with OTUs in each corresponding soil sample.*

**FIGURE 2:**
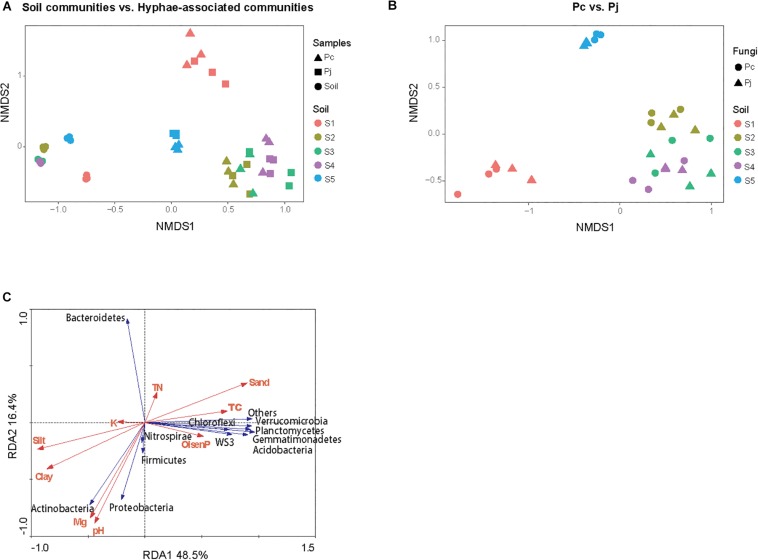
The structure of bacterial communities attached to *Penicillium* hyphae and from the bulk soil. **(A)** The differentiation of hyphae-associated bacterial communities from those of the bulk soil (S_1–5_). **(B)** Effects of soil origin on bacterial communities recruited by *P. canescens* (P_c_) and *P. janthinellum* (P_j_). The NMDS ordination was performed on OTU-based weighted UniFrac distances. **(C)** Redundancy analysis (RDA) of soil edaphic properties and the relative abundance of bacterial community associated with hyphae at phylum level.

The NMDS ordination of OTU-based weighted UniFrac distances revealed that the three top soils S_1_, S_2_ and S_5_ did not cluster, while the S_3_ and S_4_ subsoils tended to cluster with the S_2_ top soil from the same location (Figure 2A). Furthermore, it was revealed that *Penicillium* hyphae recruited bacterial communities that were significantly distinct from soil communities (PERMANOVA, *F* = 27.23, *P* = 9.99 × 10^–5^) (Figure 2A). Only around 9% of OTUs from bulk soils were present in hyphae-associated bacterial communities (Table 1). PERMANOVA based on a weighted UniFrac distance of the OTU matrix indicated that soil origin exhibited a strong and significant impact on hyphae-associated bacterial communities (*F* = 21.57, *P* = 9.99 × 10^–5^), while the effect of fungal species was not significant (*F* = 0.89, *P* = 0.45) (Figure 2B). However, a significant interaction effect was observed between soil origin and fungal species (two-way PERMANOVA, *F* = 2.06, *P* = 0.02), suggesting that the impacts of either soil origin or fungal species on recruitment were interdependent.

### Effect of Edaphic Factors on Assembly of Hyphae-Associated Bacterial Communities

Variation partitioning, based on pRDA, revealed that soil properties and fungal identities explained 57.7 and 2.7% of the total variation in hyphae-associated bacterial communities respectively. The remaining 39.6% of the variation was unexplained (Supplementary Figure S2). Mantel test further confirmed the correlation between edaphic factors distance and bacterial community dissimilarity (*P* = 0.001). As soil properties accounted for most of the observed variation in hyphae-associated bacterial communities, the effect of specific edaphic factors on relative abundances of phyla associated with hyphae was further investigated by RDA (Figure 2C). The first two axes of the RDA explained 48.5 and 16.4% of the total variation in the data. Soil pH and Mg^2+^ were positively correlated with the relative abundance of Proteobacteria (Pearson correlation coefficient: *r* = 0.74, *P* = 2.97 × 10^–6^ and *r* = 0.65, *P* = 9.16 × 10^–5^ for pH and Mg^2+^ respectively) and Actinobacteria (*r* = 0.84, *P* = 5.54 × 10^–9^ and *r* = 0.79, *P* = 1.87 × 10^–7^ for pH and Mg^2+^ respectively), and negatively correlated with the relative abundance of Bacteroidetes in hyphae-associated bacterial samples (*r* = −0.74, *P* = 2.35 × 10^–6^ and *r* = −0.71, *P* = 1.11 × 10^–5^ for pH and Mg^2+^ respectively). Other phyla including Gemmatimonadetes, Verrucomicrobia, Acidobacteria, Planctomycetes and WS3 were associated with Olsen P and TC according to RDA analysis (Figure 2C).

### Composition of Soil and Hyphae-Associated Bacterial Communities

**FIGURE 3:**
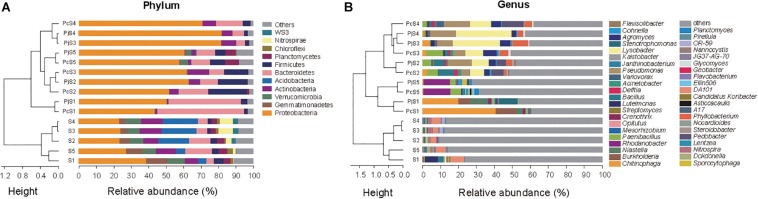
The composition of bacterial communities attached to *Penicillium* hyphae and from the bulk soil. **(A)** Relative abundance of bacterial phyla in soil and hyphae-associated samples. Community compositions were clustered based on the Bray–Curtis dissimilarity of relative abundance. “Others” represents taxa with a relative abundance less than 2%. **(B)** Relative abundance of the top 10 bacterial genera in soil and hyphae-associated samples. Community compositions were clustered based on the Bray–Curtis dissimilarity of relative abundance. “Others” represents taxa excluding the top 10 genera in either sample.

In soils, Proteobacteria was the most abundant phylum, accounting for 23–39% of all OTUs (Figure 3A). Other dominant phyla, including Gemmatimonadetes, Verrucomicrobia, Bacteroidetes, Actinobacteria, and Acidobacteria, varied significantly with soil origin (Supplementary Table S3), as also reflected in the clustering of the samples (Figure 3A). Hyphae-associated communities were also dominated by Proteobacteria, with other dominant phyla being Actinobacteria, Bacteroidetes, and Firmicutes (Figure 3A). Compared to soil communities, all the hyphae-associated bacterial communities, except for P_c__/j_S_1_, presented a significantly higher relative abundance of Proteobacteria (Supplementary Table S4). Bacteroidetes was only found to have significantly higher relative abundances in P_j_S_1_ (*P* = 1.89 × 10^–4^), and the relative abundance of Firmicutes was significantly higher in hyphae-associated communities from P_c_S_2__/__5_ (*P* = 1.13 × 10^–3^/*P* = 4.85 × 10^–4^). At the genus level, the relative abundance varied strongly with soil origin (Figure 3B). A significant increase of major genera was observed in the hyphae-associated bacterial communities as compared with the corresponding soil background. In the P_c/j_S_1_ communities, *Chitinophaga* (*P* = 0.003/*P* = 0.041 for the P_c_S_1_ and P_j_S_1_ communities respectively), *Burkholderia* (*P* = 0.002/*P* = 6.63 × 10^–7^) and *Niastella* (*P* = 0.006/*P* = 0.032) were significantly enriched, while *Rhodanobacter* (*P* = 4.57 × 10^–10^/*P* = 3.35 × 10^–5^), *Paenibacillus* (*P* = 0.007/*P* = 0.005), *Planctomyces* (*P* = 8.04 × 10^–6^/*P* = 1.98 × 10^–4^), and *Sporocytophaga* (*P* = 0.001/*P* = 3.33 × 10^–4^) were significantly enriched in the P_c/j_S_5_ communities. The relative abundance of *Phyllobacterium* (*P* = 0.033/*P* = 0.031), *Lysobacter* (*P* = 1.59 × 10^–4^/*P* = 5.19 × 10^–6^), *Paenibacillus* (*P* = 0.003/*P* = 0.02), *Agromyces* (*P* = 0.01/*P* = 0.004), and *Bacillus* (*P* = 0.02/*P* = 0.02) increased significantly in P_c/j_S_2_. P amendment to S_3_ (resulting in S_4_) had a greater influence on hyphae-associated communities than on soil communities, especially for communities attracted to P_c_ hyphae. The abundance of *Lysobacter* (*P* = 0.80), *Pseudomonas* (*P* = 0.43), *Pedobacter* (*P* = 0.12), *Delftia* (*P* = 0.49), and *Variovorax* (*P* = 0.12) in P_c_S_3_ communities was lower as compared with those in P_c_S_4_, accompanied by higher relative abundance of *Paenibacillus* (*P* = 0.18), *Agromyces* (*P* = 0.47), and *Phyllobacterium* (*P* = 0.79).

**FIGURE 4:**
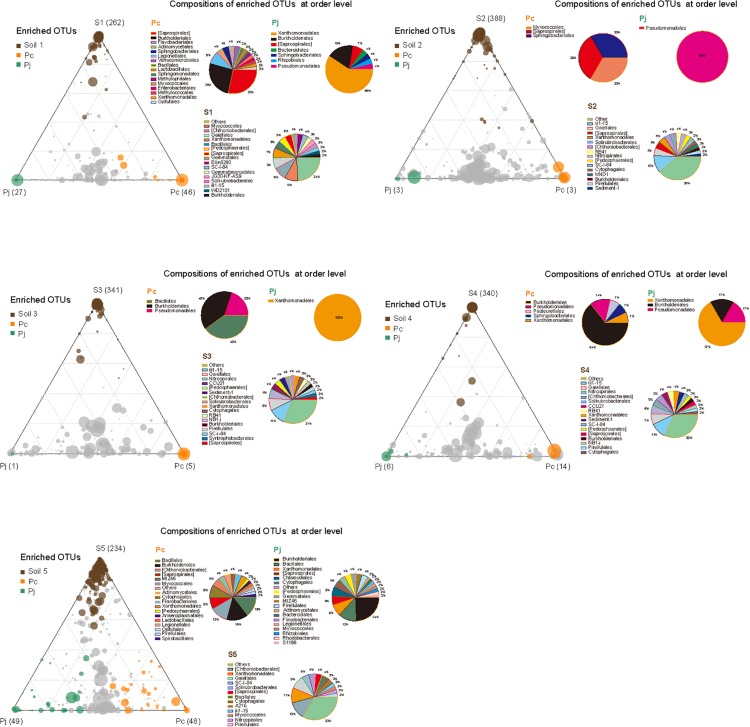
Distribution and composition of enriched OTUs for soil, *P. canescens* hyphae (P_c_) and *P. janthinellum* hyphae (P_j_) across five soil microcosms (S_1_–S_5_). A negative binomial generalized linear model was used to identify differentially enriched OTUs (RA > 0.5 ‰) for ternary plots. Circles with different colors represent OTUs significantly enriched in soil (brown), P_c_ hyphae (orange), P_j_ hyphae (green), and non-enriched OTUs (gray) respectively (FDR < 0.05). The size and position of each circle were determined by the mean relative abundance of OTUs across three compartments and the contribution of each compartment to total relative abundance respectively. Pie charts show the composition of enriched OTUs in each compartment at order level.

Although the two fungal species used in this study showed no significant difference in their overall impact on bacterial community structure, LEfSe comparisons of combined data from hyphae-associated communities across all soils showed different discriminative taxa for P_c_ and P_j_ in the same soil (Supplementary Figure S3). The selection of specific OTUs by the two fungal species was also visualized by ternary plot analyses showing that, compared to soil samples, only a small number of OTUs (less than 50 in each sample) were enriched by fungi (Figure 4). Furthermore, some enriched OTUs were only abundant in P_c_ or P_j_, indicating that each *Penicillium* species could select specific bacterial species onto their hyphae (orange and green circles in Figure 4 and Supplementary Figure S4). Taxonomic assignments for enriched OTUs at order level generally revealed a more diverse assembly in P_c_ communities than in P_j_ communities (pie charts in Figure 4). A large number of OTUs were abundant in both P_c_ and P_j_ samples, indicating a general fungal effect (gray circles in Figure 4).

### Distribution and Abundance of P-Cycling Genes in Soil and Hyphae-Associated Bacterial Communities

**FIGURE 5:**
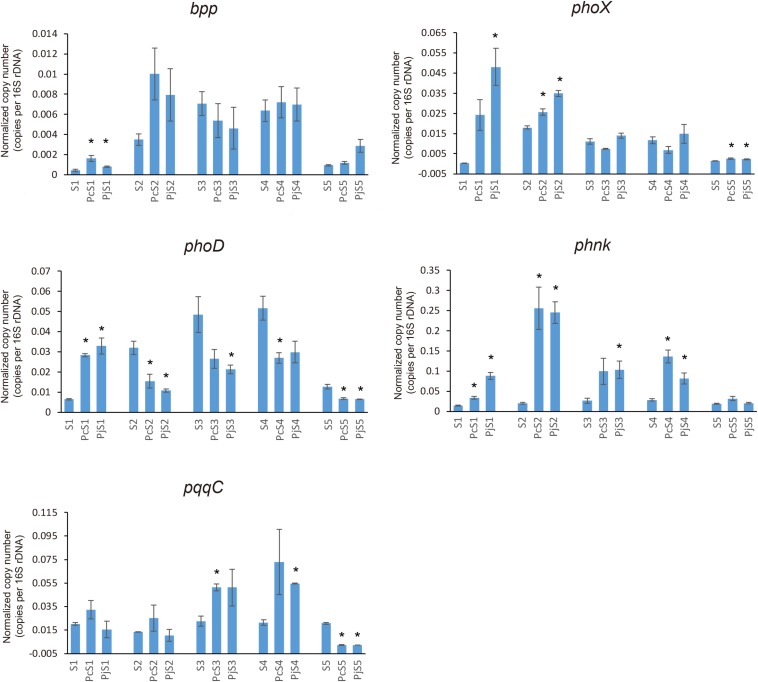
Distribution and relative abundance of phosphorus-cycling genes in soil and hyphae-associated communities. The relative abundance of genes was calculated by the ratio of absolute copy numbers of target genes to 16S rRNA genes in each sample. All data were presented as the mean ± standard error of three replicates. Significant differences in relative abundance of P-cycling genes between soil and hyphae-associated samples were analyzed in each microcosm and are indicated by stars (Student’s *t*-test, *P* < 0.05).

To determine if *Penicillium* hyphae recruit bacterial communities with a different potential for P cycling than that of soil communities, the relative abundance of selected P-cycling genes was determined by means of qPCR (Figure 5). In soil communities, *bpp*, *phoX*, and *phoD* genes varied comparably with significantly higher relative abundances in S_2_, S_3_, and S_4_ than in S_1_ and S_5_ (*P* < 0.05), while *phnK* had a significantly higher abundance in S_3_ and S_4_ as compared to S_1_, S_2_, and S_5_ (*P* < 0.05). In most hyphae-associated samples, the relative abundance of *phnK* was significantly higher than in soil (*P* < 0.05). The relative abundance of *bpp*, *phoX* and *phoD* genes showed a considerable variation between hyphae-associated samples in the different soils. Compared with soil samples, both *phoX* and *bpp* genes were more abundant in hyphae-associated samples from S_1_, S_2_, and S_5_. The *phoD* gene had significantly lower relative abundances in most hyphae-associated samples than in soil (*P* < 0.05), except for samples from S_1_ microcosms. For the inorganic P-cycling genes, only the *pqqC* gene was detectable, whereas the *ppX* gene was below the detection limit of the qPCR assay for both soil and hyphae-associated communities. The *pqqC* gene had a higher relative abundance in hyphae-associated samples as compared to the bulk soil in S_3_ and S_4_, but had a significantly lower abundance in hyphae-associated samples in S_5_ (*P* < 0.05).

Network analysis showed a shift in co-occurrence patterns between bacterial taxa and phosphorus-cycling genes from soil to hyphae-associated bacterial communities (Supplementary Figure S5). In addition, a greater diversity of potential host taxa affiliating with the *phnK* gene was observed in hyphae-associated samples as compared to bulk soil samples.

## Discussion

The significance of soil origin on the composition of hyphae-associated bacterial communities is currently not well-understood. In the present study, the application of a newly developed microcosm approach (Ghodsalavi et al., 2017) provided novel insight on the importance of soil origin as a driver for hyphae-associated bacterial community assembly, both at the taxonomic level and for the functional potential in relation to P cycling. *Penicillium*, a fungal genus that encompasses important P-solubilizing biofertilizers (Antoun, 2012), as bait, the colonization of hyphae by soil bacteria forming aggregates was observed, some of which resembled biofilms. While the ability of individual bacteria to form biofilms on hyphae is well-documented under laboratory conditions (Hogan and Kolter, 2002; Toljander et al., 2006; Brand et al., 2011), biofilm formation by bacterial communities on hyphae in soil is poorly documented (Guennoc et al., 2018).

The composition of hyphae-associated communities was distinctly different from that of the soil communities, and they were less diverse. These results are in agreement with previous studies showing shifts in bacterial communities in mycosphere soil *versus* bulk soil (Warmink and van Elsas, 2008; Gahan and Schmalenberger, 2015), however with a more pronounced decrease in diversity than previously observed. Consistently, only few phyla (Proteobacteria, Bacteroidetes, Firmicutes, and Actinobacteria) accounted for over 90% of all OTUs throughout all hyphae-associated microbiomes except the P_c/j_S_5_, where these phyla accounted for 70% of the hyphae-associated microbiomes (Figure 3A). Despite the low diversity observed, the hyphae-associated communities identified in the current study were more diverse than the community attached to *P. bilaii* hyphae in a comparable set-up, which was dominated (>90%) by Proteobacteria (Ghodsalavi et al., 2017).

Proteobacteria and Bacteriodetes are classified as copiotrophic groups which are commonly detected in carbon-enriched environments such as the rhizosphere (Yousuf et al., 2012; Aislabie and Deslippe, 2013). Here, we document the same overall recruitment of these groups to saprophytic fungi under soil conditions. When related to edaphic factors, hyphae-associated Bacteriodetes was found to correlate negatively with soil pH in the present study, while Proteobacteria and Actinobacteria were found to correlate positively with higher soil pH (*P* < 0.05). For Bacteriodetes, this correlation was mainly driven by the specific enrichment of this group in the hyphosphere of both *Penicillium* species in the low-pH soil from the Long Term Nutrient Depletion Trial, Denmark (S_1_). A strong positive correlation has previously been found between soil pH and Bacteriodetes (Lauber et al., 2009). However, in a study at the Hoosfield acid strip (Rousk et al., 2010), where pH is the main factor defining a soil gradient, no such correlation was found. The authors concluded that the often found correlation between pH and Bacteriodetes could be driven by derived factors such as C-availability. The two *Penicillium* species from the current study are known to excrete organic acids (Keromnes and Thouvenot, 1984; Zhang et al., 2002). Hence, our data support that C availability might be a stronger driver for Bacteriodetes than pH. Firmicutes may not easily be assigned to copiotrophic or oligotrophic categories (Fierer et al., 2007). They are consistently found in the plant rhizosphere (Mendes et al., 2013; Philippot et al., 2013), and as helper bacteria for mycorrhiza fungi (Frey-Klett et al., 2007; Zhao et al., 2014), where they express several beneficial traits (Frey-Klett et al., 2007; Quiza et al., 2015). They are however often found in moderate numbers. This conform with the findings of Firmicutes across all hyphae-associated communities of *Penicillium* in the present study. Despite being a dominant phylum in most hyphae-associated communities, the relative abundance of Actinobacteria declined from soil to hyphae-associated communities. This could be explained by their frequent dominance in soils with a low concentration of organic matter (Bell et al., 2014), with less ability to compete at higher nutrient levels at the hyphosphere.

A few previous studies have used baiting approaches to identify bacteria extracted from soil that are associated with hyphae under laboratory conditions. Scheublin et al. (2010) observed that Streptomycetes and members of the Oxalobacteraceae were particularly abundant on AMF hyphae formed by the genus *Glomus*. For the current study on *Penicillium*, high abundances of *Streptomyces* were found across all hyphae-associated samples, and of Oxalobacteraceae and Pseudomonadaceae in some of the samples. For Oxalobacteraceae, however, a very low abundance or absence of the genera *Collimonas*, *Massilia* and *Janthinobacterium* was found, which contrasts the observation for *Glomus* hyphae. In the current study, dominance of Oxalobacteraceae or Pseudomonadaceae in hyphae-associated microbiomes varied with soil origin. In accordance with this, members of these families have often been identified in hyphae-associated communities or in soil affected by hyphae, with varying relative abundances, possibly explained by different soil properties (Offre et al., 2007; Wang et al., 2016; Ghodsalavi et al., 2017). Taken together, our results highlight the importance of soil conditions for bacterial selection by fungi.

Bacterial communities attached to hyphae from two different *Penicillium* species with diverging ability to solubilize P under laboratory conditions, showed no significant difference in overall composition and diversity of their respective hyphae-associated microbiomes. This result is consistent with the study of Scheublin et al. (2010) on four closely-related *Glomus* isolates, while less phylogenetically-related fungi, i.e., *Trichoderma*, *Mucor* and *Rhizoctonia*, attract different hyphae-attached bacterial communities (Ballhausen et al., 2015; Rudnick et al., 2015). The composition of hyphae-associated bacterial communities may in part reflect differences in the composition of the hyphal cell walls and hyphal exudates (Rudnick et al., 2015), which would be expected to differ increasingly with a greater phylogenetic distance between fungal species. Other cases in which fungal fruiting-bodies were not directly in contact with soils showed that fungal phylogenetic variation was more important in structuring associated microbiomes than soil characteristics (Pent et al., 2018). Nevertheless, in the current study, the hyphae of individual *Penicillium* species represent unique niches for several specific hyphae-associated bacterial taxa, which could be due to the differences in fungal physiology and metabolic properties such as fungal exudate production, antibiotics production and even P solubilization capacity.

Fungal hyphae are reported as potential hotspots for nutrient cycling (Furuno et al., 2012). In this study, we observed a general enrichment of *phnK* genes in hyphae-associated bacteria from all soils, and a greater diversity of host taxa affiliated with *phnK* in the hyphae-associated microbial communities. Phosphonates can serve as a phosphorus source following bacterial C-P lyase activity, although these compounds are some of the least labile organic phosphorus (Po) sources, and their utilization has primarily been reported in marine systems (Dyhrman et al., 2009; Yu et al., 2013). Potential phosphonate sources used by hyphae-associated bacteria are components of fungal exopolysaccharides, glycoproteins and membrane phosphonolipids (McGrath et al., 2013), or phosphonate-containing antimicrobial compounds released by *Penicillium* hyphae or *Streptomycetes*, which are often abundant around hyphae (Watanabe et al., 1999; Metcalf and van der Donk, 2009). In addition to the increased potential for phosphonate degradation, a relatively high abundance of the *bpp* gene encoding a beta-propeller phytase as well as the *phoX* gene encoding a phosphatase were observed in hyphae-associated samples from topsoils with a greater organic content (S_1_, S_2_, and S_5_). Hydrolysis of phytate yields phosphoesters, which could be further hydrolyzed by a diverse group of phosphatases such as extracellular and periplasmic phosphatases (PhoX) or cytoplasmic phosphatases (PhoD) (Luo et al., 2009; Acuna et al., 2016; Neal et al., 2017). Interestingly, *phoD* genes were only enriched in the hyphae-associated community in S_1_, whereas a relative decrease was observed for all other soils. The *phoX* and *phoD* genes are important for Po recycling in rhizosphere soils (Acuna et al., 2016), but their occurrence and abundance in the hyphosphere are largely unexplored. The present study suggests a more pronounced potential ecological role of PhoX, as compared to PhoD, in the recycling of organic P around hyphae. Co-occurrence patterns identified *Pseudomonas* as the only potential host taxon affiliated with the *bpp* gene in hyphae-associated bacterial communities. This result was in accordance with a previous study showing that *Pseudomonas* is active in phytate-P turnover in the AMF hyphosphere (Wang et al., 2016). Indications for increased utilization of inorganic P in the hyphae-associated samples were less pronounced. Increased abundance of *pqqC*, one of marker genes for this process, was not consistently observed in the hyphae-associated communities as compared to the soil communities.

## Conclusion

This study demonstrated that *Penicillium* hyphae recruit distinct bacterial communities with contrasting P-cycling potential compared to the surrounding bulk soil inhabitants. The complex assemblage of bacterial communities associated with hyphae, in terms of either shifting community compositions or distribution of P-cycling genes, was mainly driven by soil origin, and only secondarily by the interactions between fungal species and soil conditions. This study highlights the importance of soil in the interaction between ecologically important fungi and their associated bacteria. The mechanisms whereby soil conditions affect hyphae-associated bacteria, however, deserve further investigation in future.

## Data Availability Statement

The datasets generated for this study can be found in the GenBank Sequence Read Archive with SRA accession: SRP132339.

## Author Contributions

MN and ON proposed the project. Y-GZ helped to design the experiments. XH performed the experiments and did data analysis. All authors participated in the manuscript writing and revising.

## Conflict of Interest

The authors declare that the research was conducted in the absence of any commercial or financial relationships that could be construed as a potential conflict of interest.

## Footnotes

1 http://huttenhower.sph.harvard.edu/lefse/
