# Supplementary material for: The Composition and Phosphorus Cycling Potential of Bacterial Communities Associated With Hyphae of Penicillium in Soil Are Strongly Affected by Soil Origin
Source: Front Microbiol. 2020 Jan 8;10:2951. doi: 10.3389/fmicb.2019.02951 (PMC6960115; doi:10.3389/fmicb.2019.02951)
Supplement: Supplementary file 1 [file Data_Sheet_1.docx]

**Supplementary Materials**

**Materials and Methods**

**Plasmid construction for functional genes involved in phosphorus cycling**

qPCR was used to quantify genes involved in phosphorus cycling, including genes encoding enzymes involved in phytic acid hydrolysis (*bpp*), phosphomonoester and phosphodiester hydrolysis (*phoD*, *phoX*), phosphonate utilisation (*phnK*), polyphosphate degradation (*ppx*), and pyrroloquinoline quinone biosynthesis (*pqqC*). Standard plasmids with the insertion of target genes were constructed using the TOPO^®^ TA Cloning^®^ Kit (Invitrogen, Carlsbad, CA, USA). Initially, the 16S rRNA gene and genes related to phosphorus cycling were amplified from the bulk soil (S_1/2_) using gene-specific primers. Primers used for all PCR reactions are listed in **Table S2**. PCR reactions were prepared in 50 µl reaction with final concentrations of 3 µM MgCl_2_, 0.5 µM dNTP, 0.2 µM each primer, 0.05 U/µl Taq DNA polymerase (Sigma-Aldrich, St. Louis, MO, USA), 1×PCR buffer without MgCl_2_ provided with the DNA polymerase, and 2 µl of DNA extract from soil samples. Thermal cycling was initiated by heating to 95 °C for 5 min, followed by 35 cycles of 95 °C for 30 s, 58 °C for 30 s, and 72 °C for 30 s, and a final extension of 72 °C for 1 min. Amplicons were purified using the QIAquick Gel Extraction Kit (Qiagen, Santa Clarita, CA, USA), cloned using the TOPO^®^ TA Cloning^®^ Kit (Invitrogen) with pCR™2.1-TOPO^®^ vector, and chemically transformed into TOP10 *E. coli* competent cells according to the manufacturer’s instructions. After verification of correct size insertions by colony PCR and gel electrophoresis, plasmids for each target gene were extracted using QIAprep Miniprep Kit (Qiagen) and sequenced to verify the identity of the insert. Copy numbers of target genes were calculated according to the plasmid DNA concentration as measured by Nanodrop, using the equation as follows:

$$Target gene (copies/\mu l)=\frac{6.02 \times{10}^{14} \times Plasmid DNA concentration (ng/\mu l)}{660 \times\left( length of vector + lenth of target gene \right)}$$
