# Supplementary material for: The Composition and Phosphorus Cycling Potential of Bacterial Communities Associated With Hyphae of Penicillium in Soil Are Strongly Affected by Soil Origin
Source: Front Microbiol. 2020 Jan 8;10:2951. doi: 10.3389/fmicb.2019.02951 (PMC6960115; doi:10.3389/fmicb.2019.02951)
Supplement: Supplementary file 2 [file Data_Sheet_2.docx]

**Supplementary Tables**

**Table S1** Characteristics of the soil used for soil microcosms

| **Soil location** | | **Soil texture (%)** | | | **pH^a^** | **Olsen P**  **(mg/kg)** | **Total C**  **(%)** | **Total N**  **(%)** | **K**  **(mg/kg)** | **Mg (mg/kg)** |
| --- | --- | --- | --- | --- | --- | --- | --- | --- | --- | --- |
|  |  | **Clay** | **Silt** | **Sand** |  |  |  |  |  |  |
| Soil 1 | LTNDT N_1_P_1_K_1_ field | 16.7 | 17.6 | 65.7 | 4.7 | 11.5 | 1.04 | 0.13 | 136 | 34 |
| Soil 2 | Sjælland Odde Org. A | 19 | 18 | 63 | 6.5 | 47.5 | 2.22 | 0.23 | 478 | 177 |
| Soil 3 | Sjælland Odde Org. B | 31 | 26 | 43 | 7.3 | 1.0 | 0.42 | 0.06 | 85 | 180 |
| Soil 4 | Soil 3 + 68 mg/kg P (NaH_2_PO_4_·H_2_O) | 31 | 26 | 43 | 7.2 | 27 | 0.42 | 0.06 | 81 | 187 |
| Soil 5 | Jyndevad A | 5 | 3 | 92 | 5.5 | 42 | 2.43 | 0.12 | 67 | 76 |

^a^ pH measured in CaCl_2_

**Table S2** Primers used in this study

| Target genes | Primers | Sequences | References |
| --- | --- | --- | --- |
| 16S rRNA gene | 341F | CCTAYGGGRBGCASCAG | ([Yu et al. 2005](#_ENREF_4)) |
|  | 806R | GGACTACNNGGGTATCTAAT |  |
| *phoD* | ALPS-F730 | CAGTGGGACGACCACGAGGT | ([Sakurai et al. 2008](#_ENREF_2)) |
|  | ALPS-R1101 | GAGGCCGATCGGCATGTCG |  |
| *phoX* | phoX2-F | GARGAGAACWTCCACGGYTA | ([Sebastian and Ammerman 2009](#_ENREF_3)) |
|  | phoX2-R | GATCTCGATGATRTGRCCRAAG |  |
| *bpp* | BPP-F | GACGCAGCCGAYGAYCCNGCNITNTGG | ([Huang et al. 2009](#_ENREF_1)) |
|  | BPP-R | CAGGSCGCANRTCIACRTTRTT |  |
| *pqqC* | pqqC2-F | AACCGCTTCTACTACCAG | ([Zheng et al. 2017](#_ENREF_6)) |
|  | pqqC2-R | GCGAACAGCTCGGTCAG |  |
| *phnK* | PhnK-F | CATCGTCGGCGAATCCGG | ([Zheng et al. 2018](#_ENREF_5)) |
|  | PhnK-R | TGCTGCATGCCGCCGGAAAA |  |
| *ppx* | ppx2-F | TGCATCTGGCGGACGGCCT | ([Zheng et al. 2018](#_ENREF_5)) |
|  | ppx2-R | AGATCCGCCGCCAATATCA |  |

**Table S3** Differences in the relative abundances (RA) of main phyla (RA > 2%) between five soil samples. One-way ANOVA was conducted followed by a Tukey’s HSD post hoc test.

| Phylum (RA > 2%) | Df | Sums of Sqs | Mean Sqs | F | P |
| --- | --- | --- | --- | --- | --- |
| Proteobacteria | 4 | 515.11 | 128.78 | 127.33 | **1.56×10^-8^** |
| Gemmatimonadetes | 4 | 164.50 | 41.13 | 238.73 | **7.11×10^-10^** |
| Verrucomicrobia | 4 | 15.21 | 3.80 | 57.65 | **7.18×10^-7^** |
| Actinobacteria | 4 | 73.65 | 18.41 | 21.71 | **6.44×10^-5^** |
| Acidobacteria | 4 | 731.61 | 182.90 | 669.87 | **4.25×10^-12^** |
| Bacteroidetes | 4 | 283.67 | 70.92 | 319.86 | **1.67×10^-10^** |
| Firmicutes | 4 | 24.91 | 6.23 | 19.87 | **9.48×10^-5^** |
| Planctomycetes | 4 | 8.40 | 2.10 | 42.34 | **3.08×10^-6^** |
| Chloroflexi | 4 | 9.88 | 2.47 | 82.32 | **1.30×10^-7^** |
| Nitrospirae | 4 | 154.26 | 38.57 | 719.49 | **2.98×10^-12^** |
| WS3 | 4 | 25.92 | 6.48 | 589.98 | **8.00×10^-12^** |
| Others | 4 | 13.44 | 3.36 | 5.20 | **1.58×10^-2^** |

Note: P values in bold mean statistical significance (P < 0.05).

**Table S4** P values of Student’s t test comparing the relative abundance (RA) of main phyla between hyphae-associated communities and their corresponding bulk soil.

| Phylum  (RA > 2%) | P_c_S_1_ | P_j_S_1_ | P_c_S_2_ | P_j_S_2_ | P_c_S_3_ | P_j_S_3_ | P_c_S_4_ | P_j_S_4_ | P_c_S_5_ | P_j_S_5_ |
| --- | --- | --- | --- | --- | --- | --- | --- | --- | --- | --- |
| Proteobacteria | 4.69×10^-1^ | 1.68×10^-2^ | 2.78×10^-4^ | 2.79×10^-5^ | 3.61×10^-4^ | 6.51×10^-4^ | 2.84×10^-4^ | 2.38×10^-6^ | 3.85×10^-6^ | 1.34×10^-5^ |
| Actinobacteria | 1.03×10^-3^ | 7.99×10^-4^ | 9.37×10^-3^ | 9.73×10^-2^ | 9.88×10^-1^ | 1.89×10^-2^ | 5.84×10^-2^ | 1.36×10^-4^ | 1.50×10^-5^ | 3.89×10^-5^ |
| Bacteroidetes | 2.12×10^-3^ | 1.89×10^-4^ | 3.01×10^-2^ | 2.82×10^-1^ | 3.16×10^-1^ | 8.44×10^-1^ | 1.40×10^-1^ | 6.27×10^-1^ | 1.27×10^-3^ | 4.47×10^-4^ |
| Firmicutes | 8.39×10^-3^ | 5.42×10^-2^ | 1.13×10^-3^ | 7.66×10^-3^ | 1.46×10^-1^ | 8.68×10^-1^ | 2.85×10^-1^ | 7.83×10^-1^ | 4.85×10^-4^ | 2.94×10^-2^ |

Note: P values in red/green represent significant higher/lower bacterial relative abundances in hyphae-associated sample than in their corresponding bulk soil (P < 0.00125 set as cut-off value due to correction for multiple testing).

**References**

Huang, H. Q., Shi, P. J., Wang, Y. R., Luo, H. Y., Shao, N., Wang, G. Z., et al. (2009). Diversity of beta-propeller phytase genes in the intestinal contents of grass carp provides insight into the release of major phosphorus from phytate in nature. Appl. Environ. Microb. 75: 6, 1508-1516.

Sakurai, M., Wasaki, J., Tomizawa, Y., Shinano, T. and Osaki, M. (2008). Analysis of bacterial communities on alkaline phosphatase genes in soil supplied with organic matter. Soil Sci. Plant Nutr. 54: 1, 62-71.

Sebastian, M. and Ammerman, J. W. (2009). The alkaline phosphatase PhoX is more widely distributed in marine bacteria than the classical PhoA. ISME J. 3: 5, 563.

Yu, Y., Lee, C., Kim, J. and Hwang, S. (2005). Group-specific primer and probe sets to detect methanogenic communities using quantitative real-time polymerase chain reaction. Biotechnol. Bioeng. 89: 6, 670-679.

Zheng, B., Zhu, Y., Sardans, J., Penuelas, J. and Su, J. (2018). QMEC: a tool for high-throughput quantitative assessment of microbial functional potential in C, N, P, and S biogeochemical cycling. Sci. China Life Sci. 61.

Zheng, B. X., Hao, X. L., Ding, K., Zhou, G. W., Chen, Q. L., Zhang, J. B., et al. (2017). Long-term nitrogen fertilization decreased the abundance of inorganic phosphate solubilizing bacteria in an alkaline soil. Sci. Rep. 7: 42284.
