# Supplementary material for: The Composition and Phosphorus Cycling Potential of Bacterial Communities Associated With Hyphae of Penicillium in Soil Are Strongly Affected by Soil Origin
Source: Front Microbiol. 2020 Jan 8;10:2951. doi: 10.3389/fmicb.2019.02951 (PMC6960115; doi:10.3389/fmicb.2019.02951)
Supplement: Supplementary file 3 [file Data_Sheet_3.pdf]

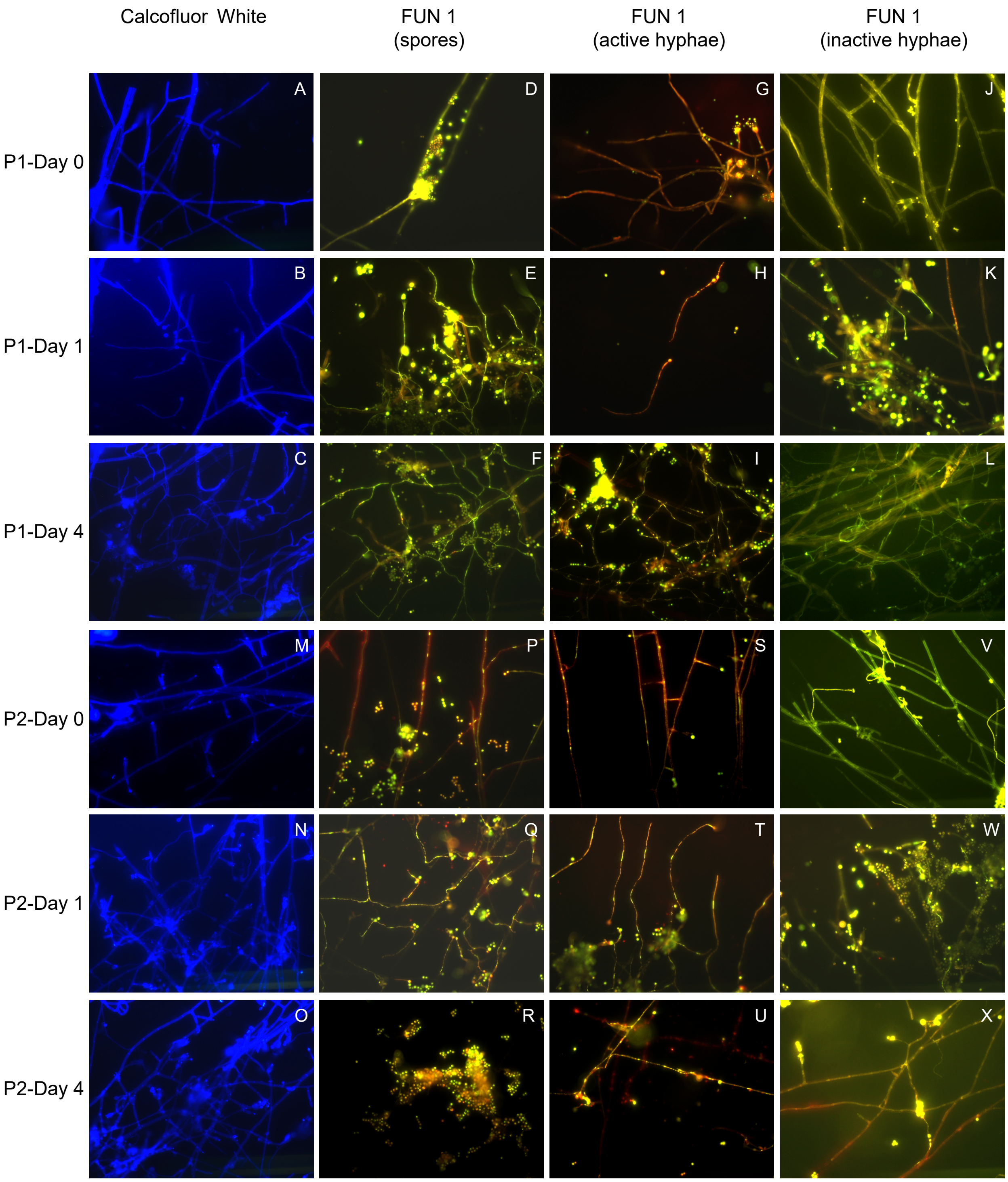

Figure S1 The structure and viability of hyphae from *P. canescens* (P_c, A-L) and *P. janthinellum* (P_j, M-X). Glass slips covered with P_c or P_j hyphae were recovered from S_1 microcosm at day 0, 1 and 4. (A-C and M-O) Calcofluor White (CFW)-stained hyphae for P_c and P_j; (D-F and P-R) mixture of both metabolically active and inactive spores for P_c and P_j; (G-I and S-U) FUN 1-stained metabolically active hyphae with red-orange intravacuolar structures for P_c and P_j; (J-L and V-X) FUN 1-stained metabolically inactive/dead hyphae with diffuse green for P_c and P_j; (J and V) negative controls using naturally killed hyphae.

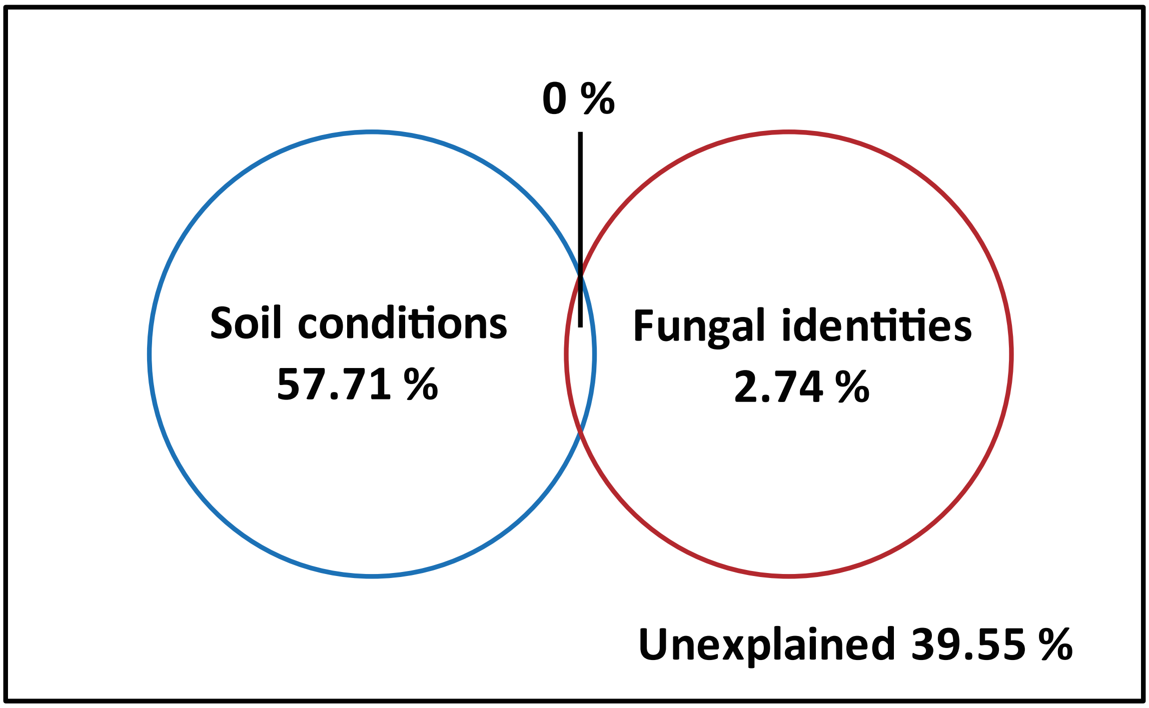

Figure S2 pRDA-based variation partitioning showing the contribution of soil conditions (soil texture, soil pH, Olsen P, total C, total N, Mg and K) and fungal identities on hyphae-associated bacterial community.

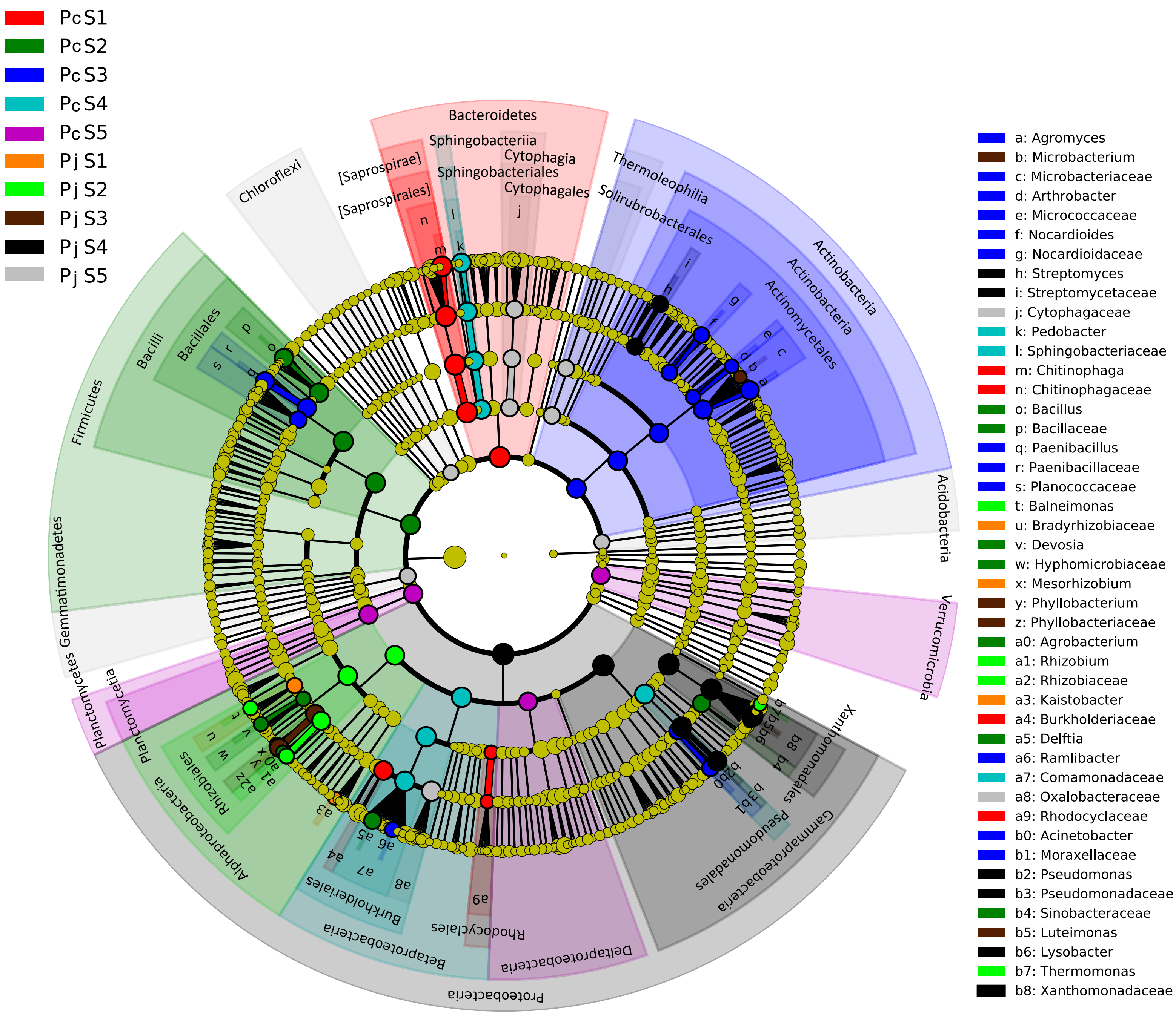

Figure S3 LEfSe (Linear discriminant analysis effect size) comparison depicting discriminating taxa among ten hyphae-associated bacterial communities ($P_{c\text{-}j}S_{1\text{-}5}$). Differentially abundant taxa were selected using Kruskal-Wallis and pairwise Wilcoxon tests ($P < 0.05$, logarithmic LDA > 2.0), and represented by different colours of the small circles and shading as taxonomic biomarkers for each group. The yellow circles on the cladogram represent taxa with non-significant differences in the relative abundance among groups. Circle size represents proportional relative abundance. Rings represent phylum, class, order, family and genus from inside to outside of the cladogram.

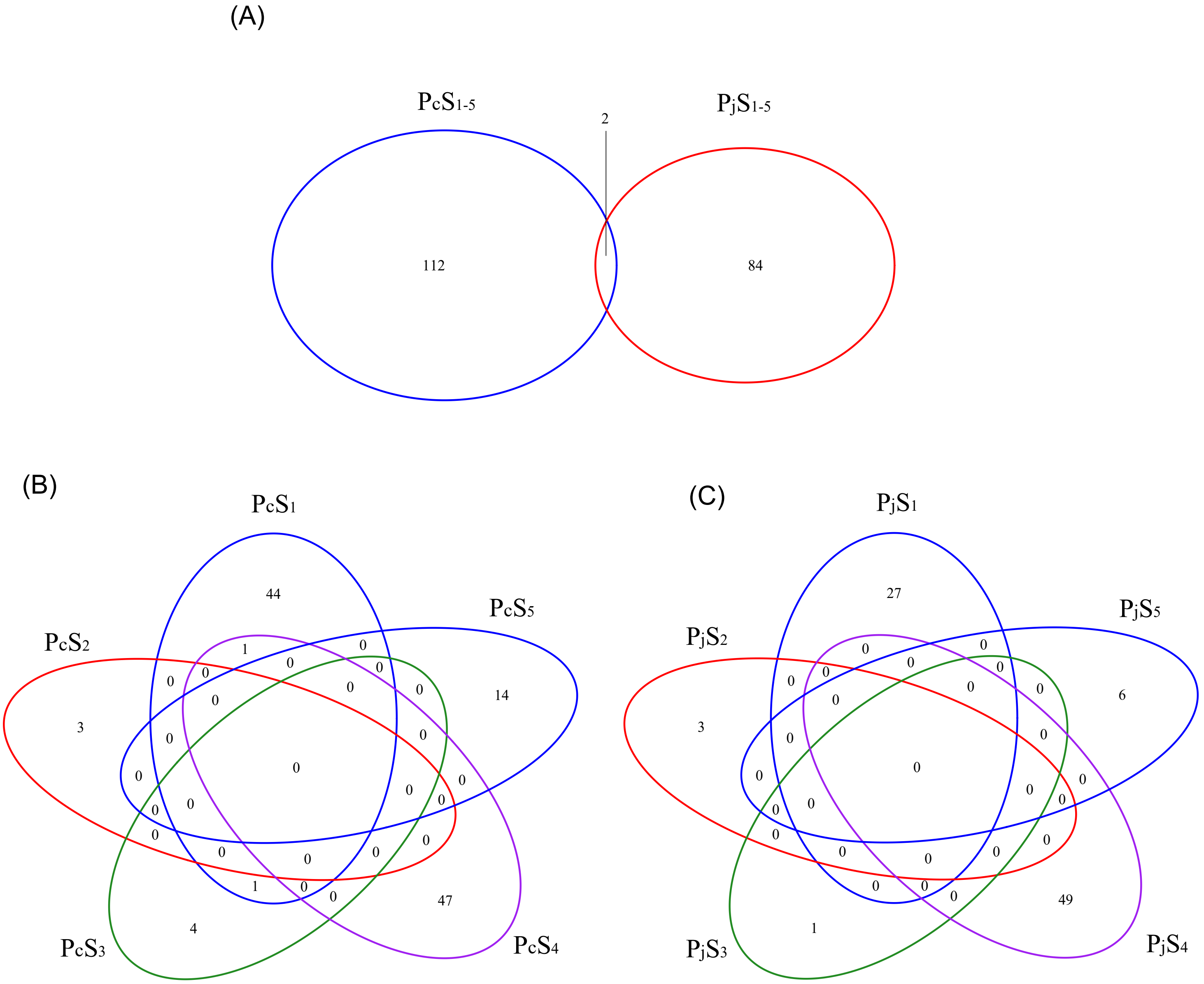

Figure S4 Venn diagrams of hyphae-enriched OTUs for *P. canescens* (P_c) and *P. janthinellum* (P_j). (A) Venn diagram of total numbers of enriched OTUs in *P. canescens* and *P. janthinellum* hyphae-associated microbiome across five soils (P_cS_{1-5} vs. P_jS_{1-5}). (B) Venn diagram of *P. canescens* hyphae-enriched OTUs in five soils. (C) Venn diagram of *P. janthinellum* hyphae-enriched OTUs in five soils.

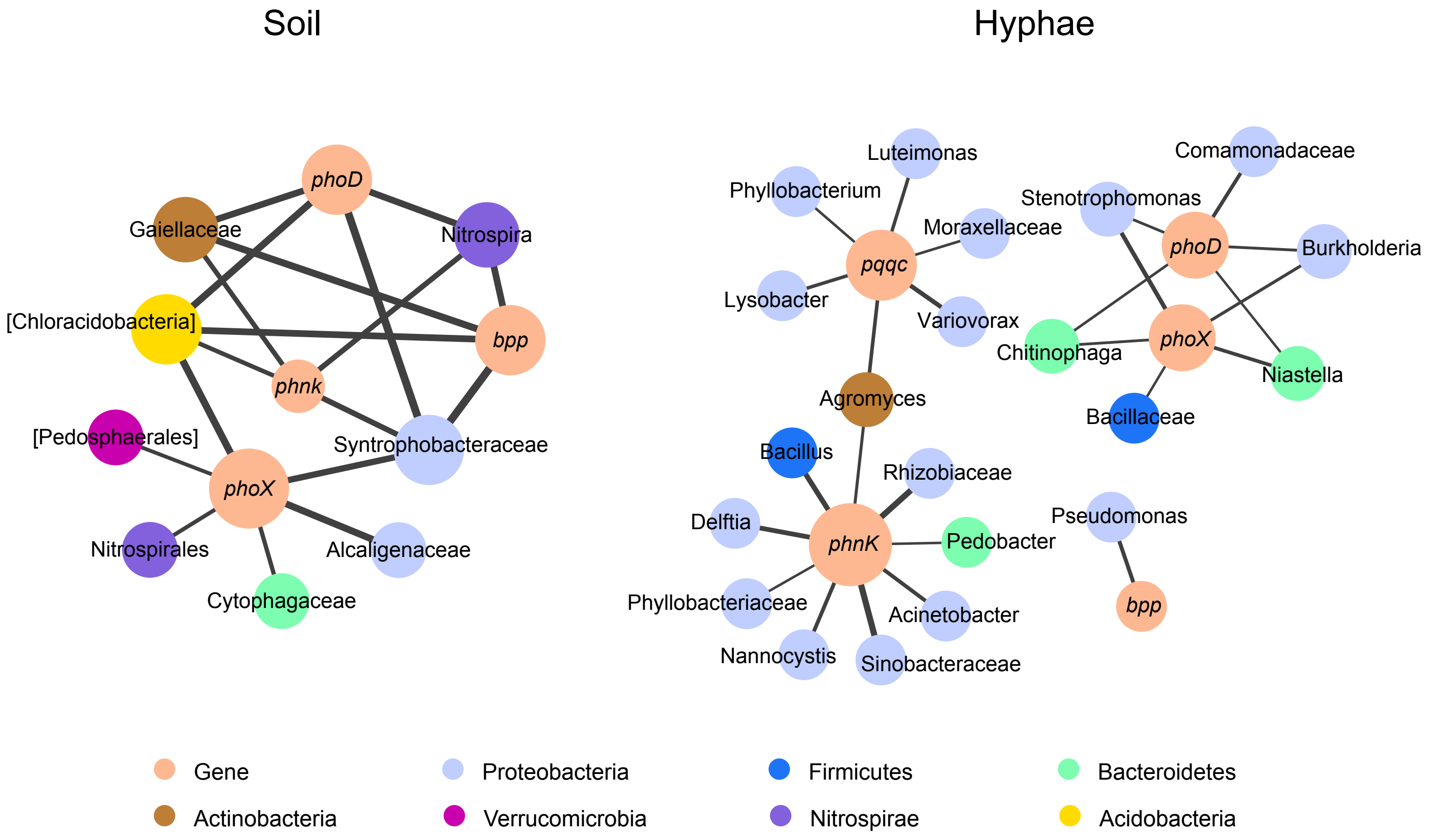

Figure S5 The co-occurrence patterns between bacterial taxa and phosphorus-cycling genes in soil and hyphae-associated samples. The nodes represent genes or bacterial taxa (coloured at phyla level), and weight according to the number of correlations to each node. A connection represents a significant ($P < 0.05$) and positive correlation according to Pearson correlation coefficients. The width of the edge is proportional to Pearson correlation coefficients.
